# Chromosomal painting of the sandpiper (*Actitis macularius*) detects several fissions for the Scolopacidae family (Charadriiformes)

**DOI:** 10.1186/s12862-020-01737-x

**Published:** 2021-01-22

**Authors:** Melquizedec Luiz Silva Pinheiro, Cleusa Yoshiko Nagamachi, Talita Fernanda Augusto Ribas, Cristovam Guerreiro Diniz, Patricia Caroline Mary O´Brien, Malcolm Andrew Ferguson-Smith, Fengtang Yang, Julio Cesar Pieczarka

**Affiliations:** 1 Laboratório de Citogenética, Centro de Estudos Avançados da Biodiversidade, ICB, Universidade Federal do Pará, PCT-Guamá, Terreno 11, Belém, Pará 66075-750 Brazil; 2 Laboratório de Biologia Molecular e Neuroecologia, Instituto Federal do Pará, Campus de Bragança, Avenida dos Bragançanos s/nº, Bragança, Pará 68600-000 Brazil; 3 Department of Veterinary Medicine, Cambridge Resource Centre for Comparative Genomics, University of Cambridge, Cambridge, CB3 0ES UK; 4 Cytogenetics Facility, Wellcome Trust Sanger Institute, Hinxton, CB10 1SA Cambridgeshire UK

**Keywords:** Chromosomal evolution, *Burhinus oedicnemus*, Classic and molecular cytogenetics, Phylogeny

## Abstract

**Background:**

The Scolopacidae family (Suborder Scolopaci, Charadriiformes) is composed of sandpipers and snipes; these birds are long-distance migrants that show great diversity in their behavior and habitat use. Cytogenetic studies in the Scolopacidae family show the highest diploid numbers for order Charadriiformes. This work analyzes for the first time the karyotype of *Actitis macularius* by classic cytogenetics and chromosome painting.

**Results:**

The species has a diploid number of 92, composed mostly of telocentric pairs. This high 2n is greater than the proposed 80 for the avian ancestral putative karyotype (a common feature among Scolopaci), suggesting that fission rearrangements have formed smaller macrochromosomes and microchromosomes. Fluorescence in situ hybridization using *Burhinus oedicnemus* whole chromosome probes confirmed the fissions in pairs 1, 2, 3, 4 and 6 of macrochromosomes.

**Conclusion:**

Comparative analysis with other species of Charadriiformes studied by chromosome painting together with the molecular phylogenies for the order allowed us to raise hypotheses about the chromosomal evolution in suborder Scolopaci. From this, we can establish a clear idea of how chromosomal evolution occurred in this suborder.

## Background

The order Charadriiformes (Aves) comprises shorebirds and is divided into three suborders: Charadrii (plovers and allies), Scolopaci (sandpipers and allies) and Lari (gulls and allies). Although cases of convergence have complicated efforts to establish their phylogenetic relationships based on morphology, the molecular phylogenies of this order have proved to be quite consistent and have given rise to few controversies [1].

The suborder Scolopaci appeared 70 million years ago and is formed by five families: Jacanidae, Rostratulidae, Thinocoridae, Pedionomidae and Scolopacidae; the latter is the most specious, with about 100 species [2]. Phylogenetically, this suborder is divided into two major branches: one leads to Scolopacidae, and one leads to the other families [2–4].

The Scolopacidae family is composed of sandpipers and snipes, which exhibit a wide distribution. These long-distance migratory birds show great diversity in their behavior and habitat use, and thus offer an important opportunity for studying the evolutionary forces that have acted on the various species [5].

Cytogenetic studies in Charadriiformes have revealed considerable karyotypic variability, with diploid numbers ranging from 2n = 42 in *Burhinus oedicnemus* [6] to 2n = 98 in *Gallinago gallinago* [7]. Compared to the members of other suborders, members of Scolopaci tend to have higher diploid numbers (Table 1), ranging from 2n = 82 in *Jacana jacana*^8^ to the previously mentioned 2n = 98 in *Gallinago gallinago* [7]. The karyotypes diverge between the two major phylogenetic branches of Scolopaci; Scolopacidae present karyotypes composed mainly of telocentric chromosomes, while in the other families most chromosomal pairs are biarmed (meta and submetacentric) [7–14].

**Table 1:** A review of cytogenetic information available for the Suborder Scolopaci

Family	Species	2n	CP	References
Jacanidae	*Hydrophasianus chirurgus*	82	–	[9]
	*Jacana jacana*	82	GGA, ZAU	[8]
	*Actitis hypoleucos*	86	–	[9]
	*Actitis macularius*	92	BOE	Present study
	*Tringa glareola*	72	–	[9]
	*Tringa totanus*	88	–	[7]
Scolopacidae	*Tringa flavipes*	88	–	[12]
	*Tringa nebularia*	88	–	[10]
	*Tringa erythropus*	88	–	[11]
	*Tringa semipalmatus*	88	–	[13]
	*Tringa ochropus*	88	–	[11]
	*Gallinago gallinago*	98	–	[7]
	*Scolopax rusticola*	88	–	[10]
	*Calidris alpina*	88	–	[10]
	*Calidris ruficollis*	86	–	[11]
	*Calidris temminckii*	90	–	[11]
	*Calidris acuminata*	84	–	[11]
	*Calidris canutus*	90	–	[11]
	*Calidris tenuirostris*	88	–	[11]
	*Arenaria interpres*	88	–	[10]
	*Limosa limosa limosa*	90	–	[14]
	*Limosa lapponica*	94	–	[11]
	*Numenius arquata*	78	–	[7]

*2n* diploid number, *FN *fundamental number, *CP* chromosome painting, species studied using whole chromosome probes, *GGA* *Gallus gallus,* *ZAU* *Zenaida auriculata*, *BOE* *Burhinus oedicnemus*

Cytogenetic studies of class Aves using chromosomal painting started with the development of whole-chromosome probes from the *Gallus gallus* macrochromosomes (GGA) [15]. These probes quickly became a reference tool in birds; their use in different orders demonstrated the recurrence of a karyotype very similar to that of GGA, leading to the proposition of a Putative Ancestral Karyotype (PAK) [16]. The PAK differs from GGA in that GGA4 is represented by two pairs in the ancestral karyotype (Table 2): PAK4 (GGA4q) and PAK10 (GGA4p). Since its proposal, the PAK has been used as a reference in comparative analyses [16–19].

**Table 2:** Chromosomal correspondence between *Gallus gallus* (GGA)*, Burhinus oedicnemus* (BOE), *Larus argentatus* (LAR), *Actitis macularius* (AMA) and *Jacana jacana* (JJA) demonstrated by chromosome painting

PAK [16]	GGA [15]	BOE [6]	LAR [20]	AMA (present work)	JJA [8]
1	1	1	1	1, 2, Wq	1
2	2	2	2	3, 11, 12, 13, Wq	4, 5p, 6p, 9
3	3	3	3	4, 14, 15, Wq	2q, 3p, 7q
4	4q	4	5	6, 16, W	2p, 3q
7, 8	7, 8	5	7, 8	7, 8	7p,6q
5	5	6	4	9, 10, Wq	5q, 8q
9	9, 2 micros (R3 and R6)	7	6, 7, 11	5, 2 micros, Wq	10
10	4p, 1 micro (R2)	8	9	?	15
6	6, 1 micro	9	6, 18	8 micros, Zq, Wq	13, 14
–	2 micros (R1 and R4)	10	4, 8	17, 20	–
–	2 micros (R2 and R7)	11	10, 16	18, 2 micros	–
–	2 micros (R5)	12	12, 17	19	20
–	2 micros (R6 and R9)	13	15, 25	6 micros, Wq	–
–	2 micros (R5)	14	13	2 micros, Wq	21
–	3 micros	15, 16	14, 19, 23	6 micros, Wq	–
–	1 micro (R9)	17, 18, 19, 20	22, 24, 26	6 micros, Wq	–
Z	Z	Z	Z	Z, Wq	Z
W	W	W	W	W, Zq	W

The numbers of chromosome pairs are the ones of the karyotype of each species

*Micro* microchromosome, *?* Hybridization did not work

In the order Charadriiformes, only four species have been studied by chromosome painting to date: (1) *Burhinus oedicnemus* (BOE, 2n = 42) has one of the lowest diploid numbers among birds (2n = 42); this is due to the fusion of many microchromosomes giving rise to macrochromosomes and made it possible (unlike the case of the GGA genome) for researchers to generate probes for all chromosomes of the karyotype [6]. (2) *Larus argentatus* (LAR, 2n = 70) was hybridized with BOE probes, which demonstrated several associations (e.g., BOE6/BOE10 and BOE7/BOE9) that were proposed as signatures for suborder Lari [20]. (3) *Vanellus chilensis* (VCH, 2n = 78) was hybridized with GGA probes; this demonstrated the fusion of GGA 7 and GGA 8, which was proposed as a possible common characteristic in suborder Charadrii [21]. (4) *Jacana jacana* (JJA, 2n = 82), which was also hybridized with GGA probes and presented numerous fissions and fusions, demonstrating that JJA had undergone extensive genomic reorganization [8].

**Fig. 1:**
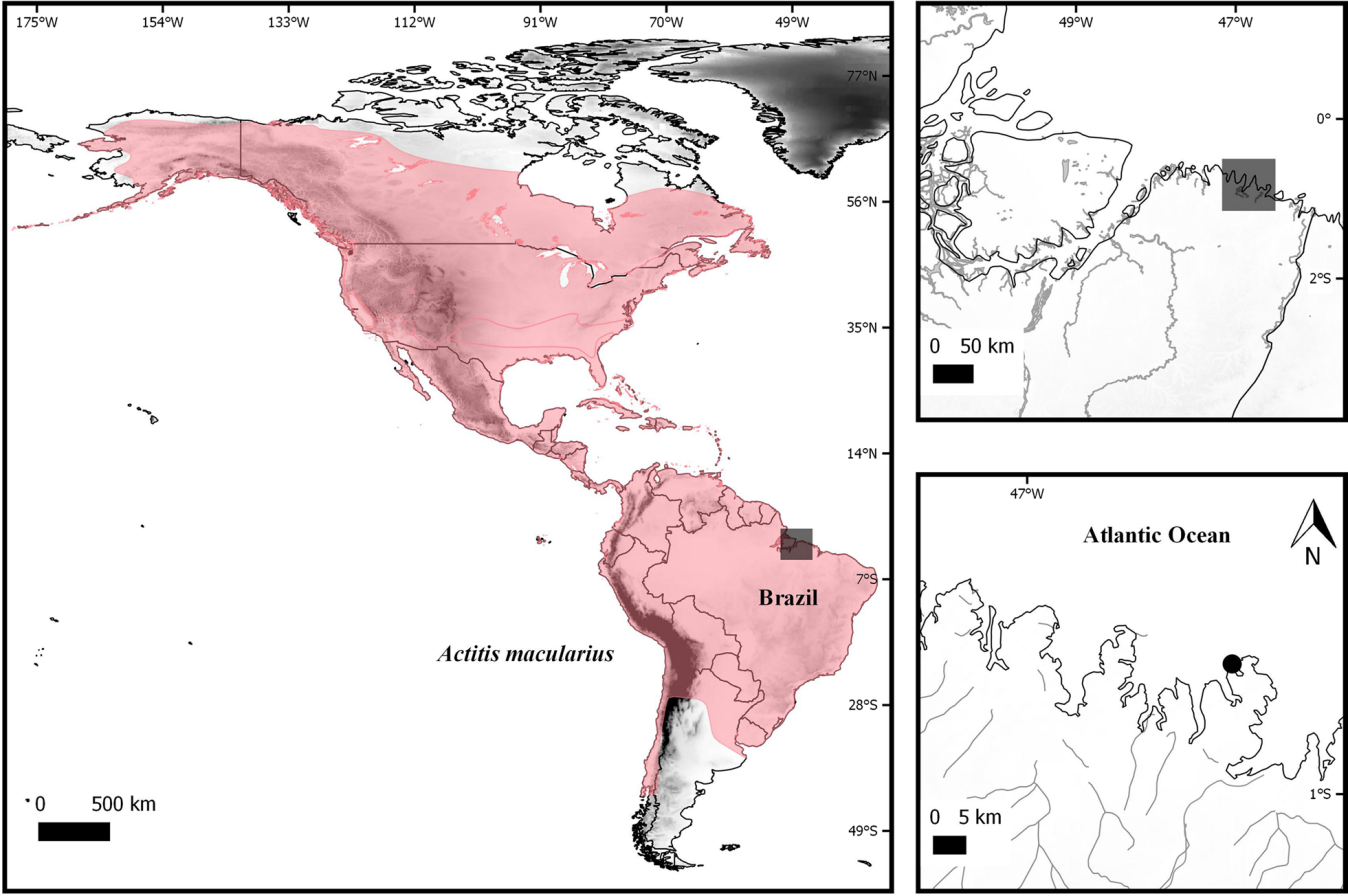
Geographic distribution map showing the collection locality (Bragança—PA) and place from which the *Actitis macularius* specimens were collected. Specimens were collected at Praia do Pilão (black circle). The map was prepared using the QUANTUM-GIS software, v. 2.10.1. The database was obtained through IBGE and REDLIST

In sum, there is relatively little cytogenetic information described so far for Charadriiformes, but there is considerable chromosomal variation between the different taxonomic groups (Fig. 1). Thus, further studies are needed to improve our understanding of the karyotypic evolution of these families. In the present study, we analyzed the karyotype of *Actitis macularius* (AMA, Scolopacidae) using BOE probes [6] and compared it with published data for *Gallus gallus* (GGA) and Charadriiformes. We also revised the cytogenetic information available for the suborder Scolopaci, with special emphasis in families Jacanidae and Scolopacidae (Table 1), and used a published molecular phylogeny [2] to determine the direction of chromosomal rearrangements.

## Results

### Karyotypic description and chromosome painting in *Actitis macularius*

**Fig. 2:**
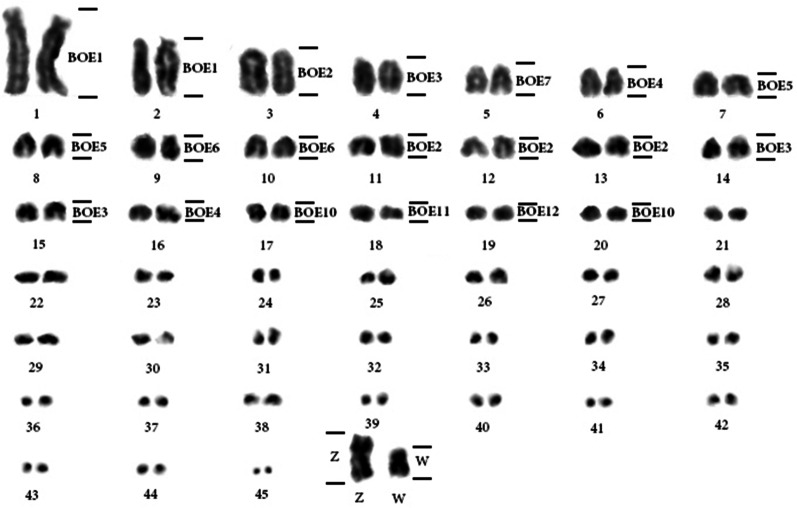
*Actitis macularius* karyotype with genomic mapping performed using *Burhinus oedicnemus* (BOE) whole-chromosome probes, with the correspondence shown on the right. The microchromosomes were organized by size, as the correct homologies could not be detected due to the lack of reliable markers

*Actitis macularius* has 2n = 92, where the first two pairs are acrocentric and the others are telocentric (Fig. 2). This karyotype has 14 pairs of autosomal macrochromosomes and the others are microchromosomes. For the sex chromosomes, the Z and the W are submetacentric.

**Fig. 3:**
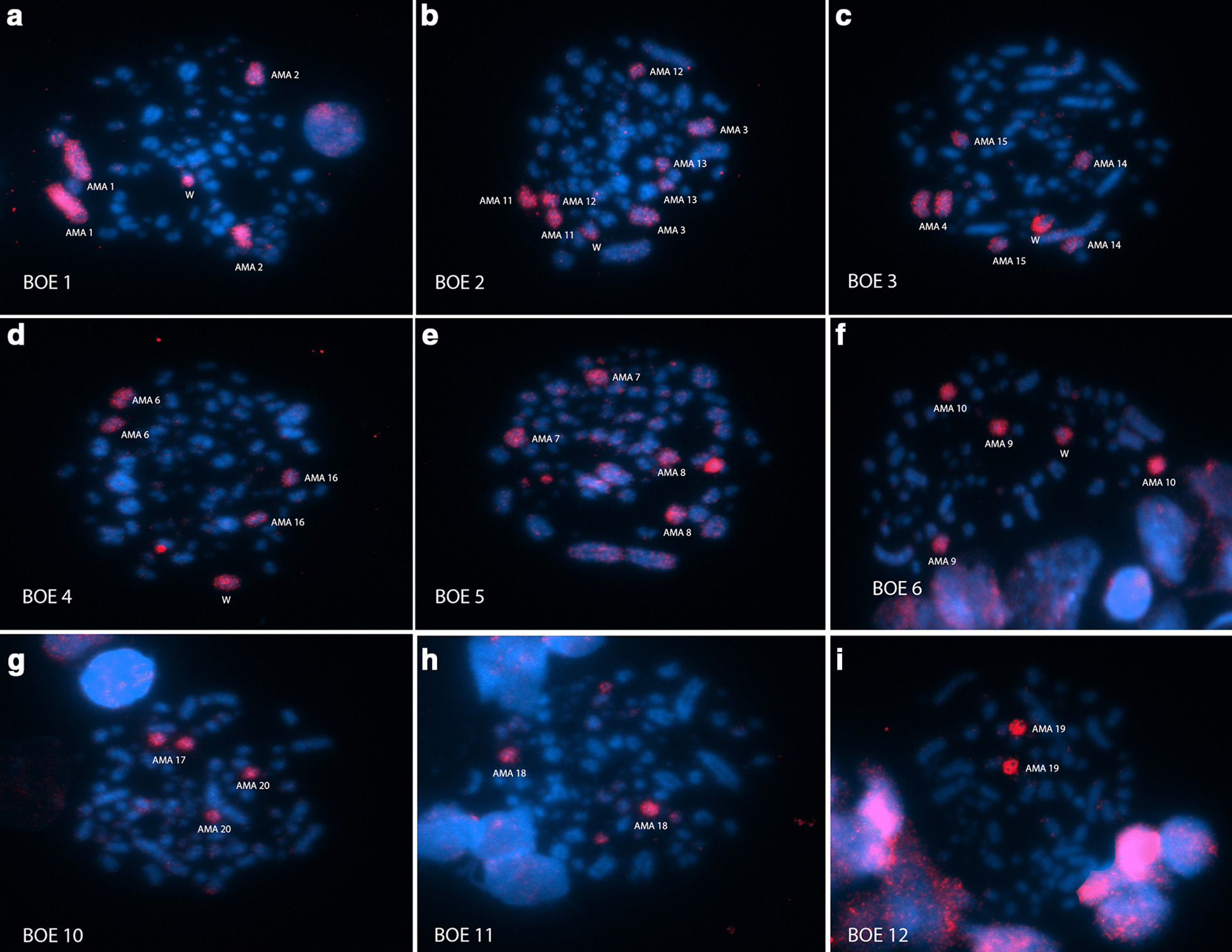
Chromosome painting with whole-chromosome probes from *Burhinus oedicnemus* (BOE) in *Actitis macularius* (AMA). **a** BOE1 (pairs 1, 2 and W); **b** BOE2 (pairs 3, 11, 12, 13 and W); **c** BOE3 (pairs 3 and 14, 2 microchromosomes and W); **d** BOE4 (pair 6, 2 microchromosomes and W); **e** BOE5 (pairs 7 and 8 and W); **f** BOE6 (pairs 9 and 10 and W); **g** BOE10 (pairs 17 and 20); **h** BOE11 (pair 18 and a pair of microchromosomes); **i** BOE12 (pair 19). The probes were visualized with avidin-Cy3 (red) and the chromosomes were counterstained with DAPI (blue)

FISH with BOE whole-chromosome probes in *Actitis macularius* (AMA) demonstrated the correspondences observed in Fig. 2 and Table 2. Examples of these hybridizations are found in Fig. 3. These results were extrapolated to GGA and PAK using the previous reports [6, 16], respectively (Table 2).

## Discussion

### The karyotype of *Actitis macularius* (AMA)

The karyotype of AMA, which was studied herein for the first time, has 2n = 92, and thus exhibits a 2n greater than the proposed 2n = 80 for the PAK [16]. This difference reflects the occurrence of fission rearrangements in all macrochromosomes. As this is a common feature among Scolopaci (Table 1), this is not a distinctive feature specific of *Actitis macularius*.

### Chromosomal rearrangements among* Actitis macularius* (AMA), *Burhinus oedicnemus* (BOE) and *Gallus gallus* (GGA)

Using BOE probes to paint the karyotype of a species of Scolopacidae allowed us to detect the rearrangements that occurred in the phylogenetic branch leading to the AMA karyotype. Unlike the conserved state observed for the first pairs of many avian species [16, 19, 22, 23], including *Burhinus oedicnemus* [6], pairs 1, 2, 3, 4 and 6 of PAK are fissioned in AMA. Possibly other Scolopacidae with high 2n and similar chromosomes may have undergone the same rearrangements.

Many BOE probes hybridized on the long arm of the W in AMA, as observed in *Larus argentatus* [20]. This suggests that the W carries numerous variable copies (repetitive regions) homologous to the autosomes of species in order Charadriiformes. A similar arrangement was found in the Passeriform, *Glyphorynchus spirurus* [24]. An experiment to test the possibility of repetitive DNA sequences spread in autosomes and W would be the isolation of this sequence and its use as a DNA probe for FISH in AMA karyotype.

**Fig. 4:**
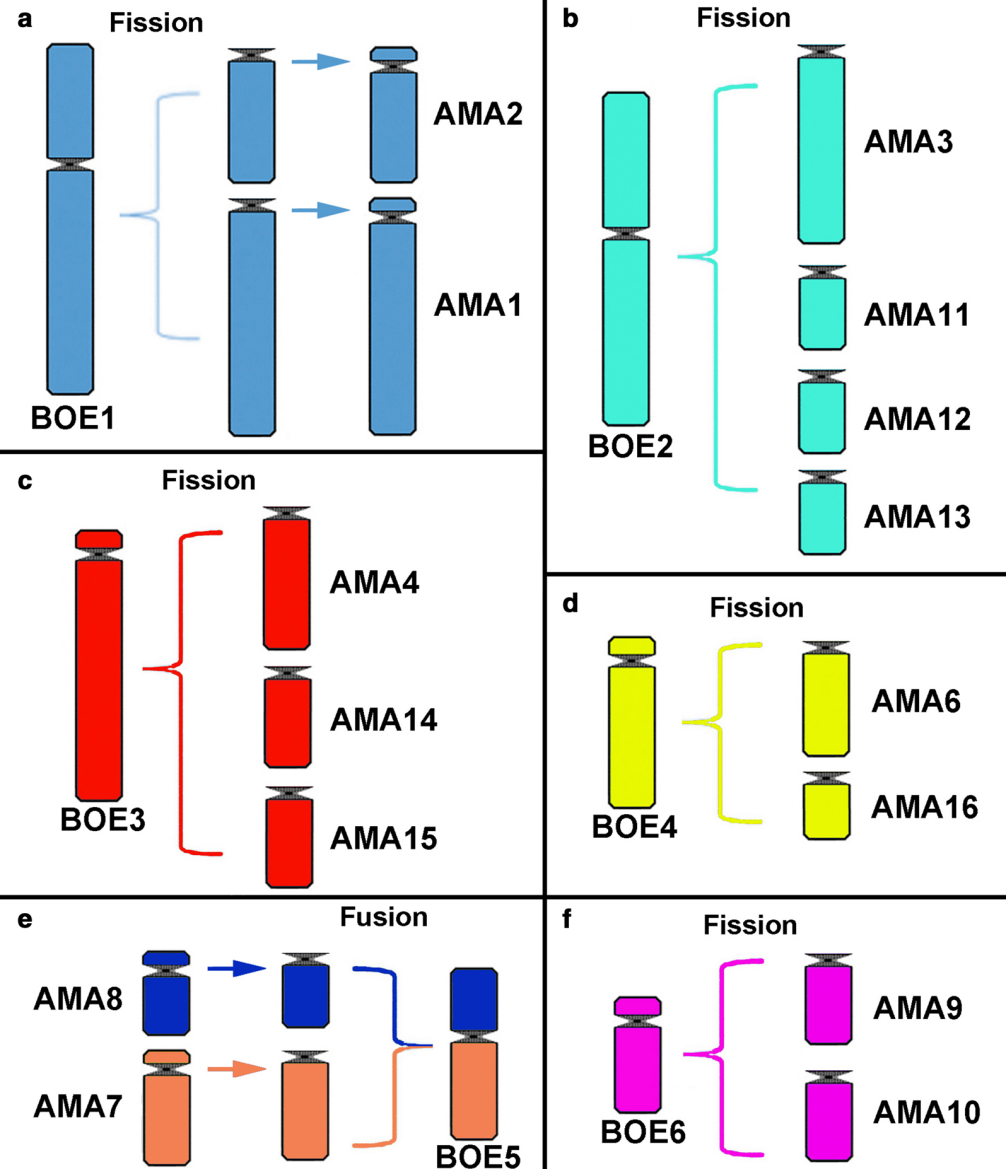
Idiogram of the rearrangements found in our comparison of the BOE and AMA karyotypes. The colors correspond to the PAK idiogram (Fig. 5). Fission occurred in: **a** BOE1 (= PAK1), forming AMA1 and AMA2; **b** BOE2 (= PAK2), generating AMA3 and AMA11, 12 and 13; **c** BOE3 (= PAK3), originating AMA4, 14 and 15; **d** BOE4 (= PAK4), generating AMA6 and 16; **e** BOE5 (= PAK7, 8), yielding AMA7 and 8; and **f** BOE6 (= PAK5), forming AMA9 and 10

Despite BOE belonging to the same order as AMA, we observed no conservation of the macrochromosome pairs, except for the Z and W. The fission of submetacentric BOE1 is clear in the first two AMA acrocentric pairs. Pericentric inversion may have occurred after this fission, leading to the formation of two pairs with small short arms (Fig. 4a). Alternatively, the short arm may be the result of telomeric amplification [25] or centromeric repositioning [26]. The submetacentric BOE2 is divided into four telocentric pairs in AMA (pairs 3 and 11–13) due to multiple fissions; we were not able to define which segment of BOE2 was found in each AMA pair (Fig. 4b). BOE3 experienced fission, giving rise to pairs AMA4, 14 and 15 (Fig. 4c). Fission of BOE4 gave rise to AMA6 and AMA16 (Fig. 4d). BOE5 was divided into two pairs, AMA7 and AMA8, but this was not by fission. Cytogenetic studies demonstrated that the fusion of PAK7 and PAK8 is a characteristic of suborder Charadrii [21]; the presence of the separate chromosomal pairs in *Actitis* is the ancestral form, and BOE5 is the derived form (Fig. 4e). Fission of BOE6 gave rise to AMA9 and 10 (Fig. 4f). BOE8 did not hybridize in the AMA genome, possibly for technical reasons. So, this is first confirmation of the fissions in pairs BOE2, 3, 4 and 6 and also the first demonstration of fission in pair BOE1 in Scolopaci.

Fissions in BOE1, 2 and 5, were observed in *Glyphorynchus spirurus* [24], Strigiformes, Passeriformes, Columbiformes and Falconiformes also have the fission of GGA1 (BOE1) [20, 27–29]. For Scolopaci, in contrast, a fission of PAK1 (= GGA1, BOE1) seems to be a character shared only among members of the Scolopacidae family. Its presence in other orders would therefore be an example of homoplasy.

The correspondences among the AMA, BOE, LAR, GGA and PAK chromosomes are shown in Table 2.

### Chromosome evolution in suborder Scolopaci

It is accepted that the ancestral putative karyotype (PAK) with 2n = 80, which is commonly found in several orders of birds, remained conserved for about 100 million years, with few variations for Neoaves [16]. However, order Charadriiformes presents a high level of karyotypic diversity [14]. An interesting point is that the three suborders originated in the late Cretaceous between 79 and 102 Mya [3], which indicates that little time has passed from the origin of PAK to the ancestral Charadriiformes karyotype.

**Fig. 5:**
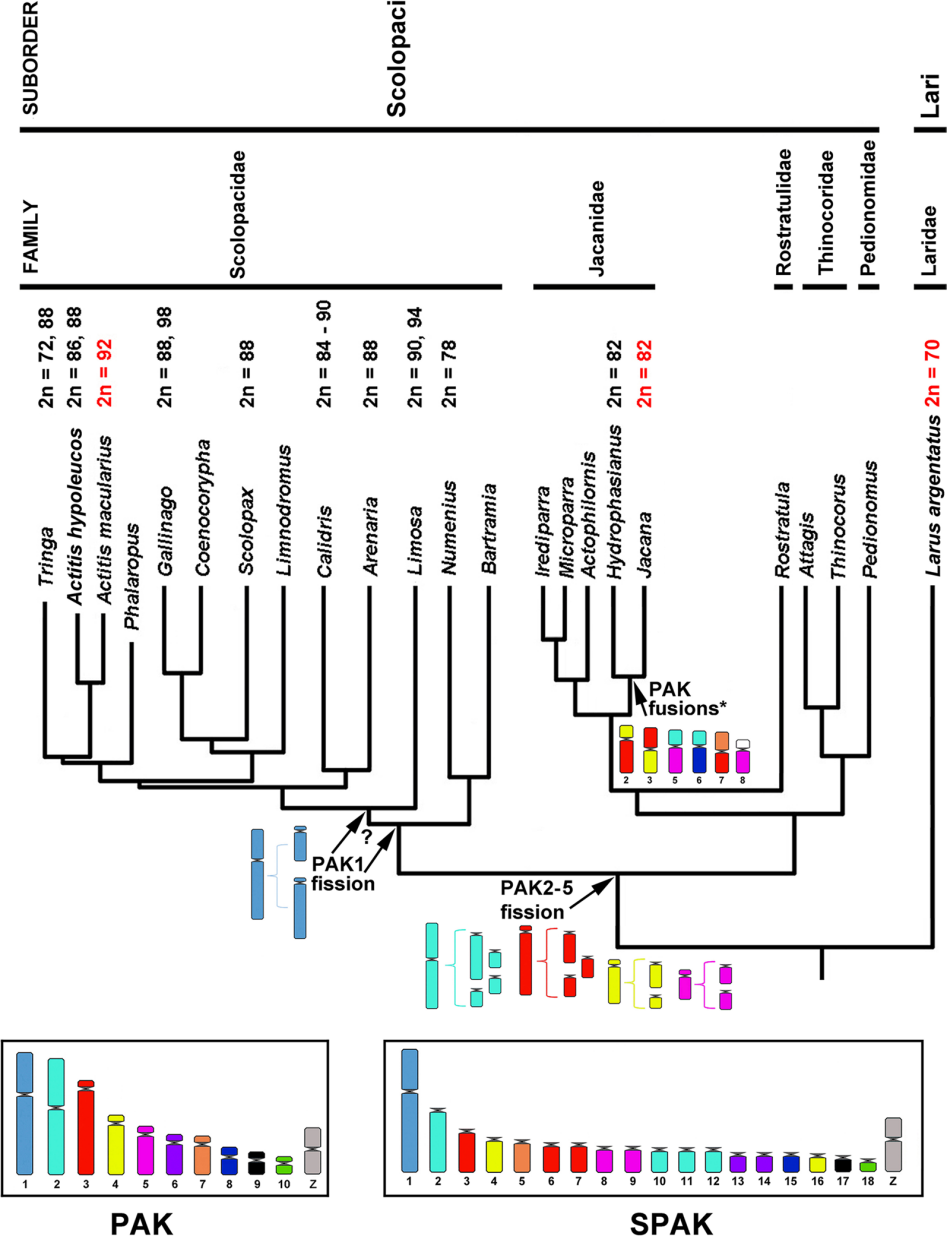
A simplified version of the phylogeny from Gibson and Baker [2], which was based on the sequences of five genes (RAG1, CYT B, 12S, ND2 and COI) and estimated with partitioned Bayesian analysis for suborder Scolopaci. According to the authors, “all nodes received a posterior probability of 1.00 unless otherwise labeled”. In the partial phylogeny here shown, the only node that has a posterior probability lower than 1.00 is the one that split *Actitis* from *Tringa* (0.71). Diploid numbers from literature (Table 1); karyotypes analyzed by FISH are shown in red. PAK fusions* = (4 + 3), (3 + 4), (2 + 5), (2 + 8), (7 + 3), (5 + micros), according to Kretschmer et al. [8]. *PAK* avian putative ancestral karyotype, Griffin et al. [16]. *SPAK* Scolopaci PAK

Suborder Scolopaci has a high diploid number, ranging from 78 to 98 chromosomes [9]. In addition to *Actitis macularius* (described here), chromosome painting was previously used to examine the karyotype of *Jacana jacana* [8]. Our comparative analysis between these two species (Table 2) shows that both share the following fissions: PAK2 (GGA2, BOE2) in four segments; PAK3 (GGA3, BOE3) in three segments; and PAK4 (GGA4q, BOE4) and PAK6 (GGA6, BOE9) in two segments. After these fissions, a series of fusions occurred between several chromosome pairs in the evolutionary branch that gave rise to JJA [8]. Although the literature lacks any additional chromosome painting study of the Jacanidae, the karyotype described from the other genus of this family, *Hydrophasianus*, has the same diploid number and appears similar to JJA on conventional staining [9]. This suggests that the chromosomal characteristics found in JJA are not restricted to this species and may be chromosome signatures for the Jacanidae family (Fig. 5).

An interesting feature is seen for chromosome PAK1 (= BOE1, GGA1): It is split into two pairs in AMA but remains whole in JJA. This suggests that PAK1 underwent fission in the branch that led to AMA but remained in its ancestral form in JJA. Since the morphology of the full-length PAK1 chromosome is quite different from that of its split version, information about the timing of this fission can be obtained by analyzing other karyotypes along the branch that leads to AMA (family Scolopacidae), even using conventional staining data. The karyotypes of genera *Tringa*, *Calidris*, *Arenaria* and *Limosa* [7, 9–11, 13, 14] show the first chromosomal pair as an acrocentric that is similar in size to the long arm of PAK1, according to the measurements performed by Hammar [7]. This suggests that fission occurred in the branches that lead to these genera (Fig. 5). Two branches cast some doubt on this proposition, however. The *Gallinago* and *Scolopax* genera have similar karyotypes, in which the first pair is a submetacentric chromosome [7, 10, 13]. Hammar [7] measured the chromosomes of several species of Charadriiformes and demonstrated that the first whole-chromosome pair (PAK1) is equivalent to 14% of the karyotype. In contrast, that of *Tringa* (long arm of PAK1) corresponds to 10%. The first pair of the *Gallinago* karyotype corresponds to 9.3% of the karyotype, suggesting that it is similar to *Tringa*, but with an inversion. Chromosomal painting studies are needed in these species to confirm if the first pair of *Tringa* and *Gallinago* are homeologues. The second branch that generates doubt is the one that leads to *Numenius* and *Bartramia* (Fig. 5). The second chromosomal pair of the karyotype of *Numenius arquata* is an acrocentric corresponding to 9.8% of the karyotype [7]; it may be equivalent to the first pair of the other species of Scolopaci (the first pair of the karyotype of *Numenius* is a metacentric of similar size, possibly the result of a fusion). Studies with chromosome painting in *Numenius* and/or *Bartramia* are needed to test this possible correspondence. Thus, it is not clear whether the fission break in PAK1 occurred at the base of the branch that gave rise to the Scolopacidae family or after the separation of the branch that gave rise to *Numenius* and *Bartramia* (Fig. 5). If additional studies confirm that *Numenius arquata* pair 2 is equivalent to the first chromosome of the other species of Scolopacidae, the fission of PAK1 would be a chromosomal signature for this family.

The rearrangements described here are restricted to suborder Scolopaci, since chromosomal painting in *Larus argentatus* [20], a species of suborder Lari (a sister group of Scolopaci) [1, 4], revealed a karyotype similar to the ancestral birds in pairs PAK1-4, with fusions of microchromosomes with macrochromosomes (LAR4 and 7–9) and none of the fissions observed in Scolopaci (Table 2).

The data analyzed here allow us to propose an ancestral karyotype for suborder Scolopaci using PAK as an outgroup, in which chromosome PAK1 is preserved, PAK2 is broken into four pairs, PAK3 is fragmented into three segments and PAK4 and PAK6 are divided into two segments each (Scolopaci Putative Ancestral Karyotype, SPAK, Fig. 5).

## Conclusions

This study examined for the first time a species of family Scolopacidae by classic and molecular cytogenetics. *Actitis macularius* has a high diploid number with several fissions. This work suggests that fissions and possible pericentric inversions occurred in suborder Scolopaci, leading to a karyotype formed almost exclusively of telocentric pairs. Only five species have been examined to date with *Burhinus oedicnemus* probes, but the data from the previous and present studies enable us to establish a clear idea of how chromosomal evolution occurred in this suborder. Studies of more species are needed to test the hypotheses raised here and further clarify the evolutionary history of this group of birds.

## Methods

### Ethics

The specimens were kept stress-free with full access to food and water until their necessary euthanasia, which was performed by intraperitoneal injection of buffered and diluted barbiturates (86 mg/kg) after anesthesia with ketamine (40 mg/kg), following The American Veterinary Medical Association Guidelines for the Euthanasia of Animals, in accordance with animal welfare guidelines established by Brazilian resolution CFMV n.1000/2012, and with animal welfare guidelines established by the Animal Ethics Committee (Comitê de Ética Animal) from Universidade Federal do Pará (UFPA), which authorized the present study (Permit 68-2015). JCP has a permanent field permit, number 13248 from “Instituto Chico Mendes de Conservação da Biodiversidade”. The Cytogenetics Laboratory from UFPA has a special permit number 19/2003 from the Ministry of Environment for samples transport and 52/2003 for using the samples for research.

### Sampling

Three *Actitis macularius* adult females were collected during field research for the Molecular Biology and Neuroecology Laboratory of the Federal Institute of Pará (Laboratório de Biologia Molecular e Neuroecologia do Instituto Federal do Pará), Campus Bragança, and so they provided all technical support. Collections occurred at Praia do Pilão (0º 47′ 46.08ʺ S and 46° 40′ 29.64ʺ W) in the state of Pará, Brazil (Fig. 1). Nets of 12 m × 2 m in size and made with a 36-mm mesh were extended, and then visited every 30 min for sample retrieval [30]. Voucher specimens (BCAM108, BCAM109 and BCAM126) were deposited in the collection of the Laboratório de Biologia Molecular e Neuroecologia, Instituto Federal do Pará (Bragança, Para, Brazil).

### Cytogenetic methods

#### Chromosome preparation and classic cytogenetics

As the sample would be euthanatized for other research purposes not related to cytogenetics, bone marrow preparations were performed in the field after colchicine treatment according to the literature [31].

Mitotic chromosomes were classified in decreasing sizes according to the proposed nomenclature [32]. The metaphases were captured and the karyotypes were assembled using the Adobe Photoshop CS6 software.

#### Chromosome painting

The utilized whole-chromosome probes from *Burhinus oedicnemus* (Charadriiformes) were obtained by flow cytometry [6]. The fluorescence in situ hybridization (FISH) experiments were carried out as described by Yang [33]. Metaphase chromosome preparations were made and aged at the same day for 1 h at 65 °C, followed by incubation in 1% pepsin for 5 min. These slides preparations were denatured in 70% formamide, 2×SSC solution at 65 °C for 1 min, rapidly cooled in ice-cold 70% ethanol and dehydrated through a 70%, 90%, and 100% ethanol series. The probes (1 μl) were diluted into 15 μl of hybridization buffer (50% deionized formamide, 10% dextran sulphate, 2×SSC, 0.5 M phosphate buffer, pH 7.3), denatured at 65 °C for 10 min, and applied onto slides, followed by a three days hybridization at 42 °C. After hybridization the preparations were washed twice in formamide 50%, 2×SSC, and once in 4×SSC/Tween at 40 °C. For visualization of the biotin-labelled probes a layer of Cy3-a or Cy5-avidin (1:1000 dilution; Amersham) was used. For FITC-labelled probes we used a layer of rabbit anti-FITC (1:200; DAKO). Slides were mounted in a mounting medium with DAPI called Vectashield (Vector Laboratories). For the first mapping single experiments (one probe by slide) were made. For the determination of the limits of two different probes into a chromosome pair, double experiments (two probes using different colors) were made.

Slides were analyzed in a Nikon H550S microscope, with a DS-Qi1Mc digital camera controlled by the Nis-Elements software. The images were captured in black and white and subsequently pseudo-colored based on the utilized fluorochromes. Images were edited with the Adobe Photoshop CS6 software.

### Phylogenetic inferences

To determine the direction of chromosomal rearrangements in Scolopaci, the known karyotypes were plotted on a molecular phylogeny [2]. The phylogeny was built by those authors based on the sequences of five genes (RAG1, CYT B, 12S, ND2 and COI) and estimated with partitioned Bayesian analysis (Fig. 5). To make the chromosomal evolution clear, we redesigned the phylogeny, where branches with genera without cytogenetic information were removed, but at least one representative from each family remained. We also take into account the PAK as an outgroup to define the direction of the rearrangements (fusion or fission).

## Data Availability

All data supporting the results reported in this article can be found at the article itself. No additional dataset is available.

## Abbreviations

GGA: *Gallus gallus*
PAK: Putative ancestral karyotype
BOE: *Burhinus oedicnemus*
2n: Diploid number
LAR: *Larus argentatus*
VCH: *Vanellus chilensis*
JJA: *Jacana jacana*
AMA: *Actitis macularius*
FISH: Fluorescence in situ hybridization
